# Adverse events of vaccines and the consequences of non-vaccination: a critical review

**DOI:** 10.11606/S1518-8787.2018052000384

**Published:** 2018-04-04

**Authors:** Luana Raposo de Melo Moraes Aps, Marco Aurélio Floriano Piantola, Sara Araujo Pereira, Julia Tavares de Castro, Fernanda Ayane de Oliveira Santos, Luís Carlos de Souza Ferreira

**Affiliations:** I Universidade de São Paulo. Instituto de Ciências Biomédicas. Departamento de Microbiologia. São Paulo, SP, Brasil; II Instituto Butantan. Centro de Biotecnologia. São Paulo, SP, Brasil; III Instituto Adolfo Lutz. Centro de Imunologia. São Paulo, SP, Brasil

**Keywords:** Vaccination, utilization, Vaccination, adverse effects, Vaccines, Immunization, Adjuvants, Immunologic, contraindications, Risk Factors, Communicable Disease Control, Vacinação, utilização, Vacinação, efeitos adversos, Vacinas, Imunização, Adjuvantes Imunológicos, contraindicações, Fatores de Risco, Controle de Doenças Transmissíveis

## Abstract

**OBJECTIVE:**

To analyze the risks related to vaccines and the impacts of non-vaccination on the world population.

**METHODS:**

This is a narrative review that has considered information present in the bibliographic databases NCBI-PubMed, Medline, Lilacs, and Scientific Electronic Library Online (SciELO), between November 2015 and November 2016. For the analysis of outbreaks caused by non-vaccination, we considered the work published between 2010 and 2016.

**RESULTS:**

We have described the main components of the vaccines offered by the Brazilian public health system and the adverse events associated with these elements. Except for local inflammatory reactions and rare events, such as exacerbation of autoimmune diseases and allergies, no causal relationship has been demonstrated between the administration of vaccines and autism, Alzheimer's disease, or narcolepsy. On the other hand, the lack of information and the dissemination of non-scientific information have contributed to the reemergence of infectious diseases in several countries in the world and they jeopardize global plans for the eradication of these diseases.

**CONCLUSIONS:**

The population should be well informed about the benefits of vaccination and health professionals should assume the role of disseminating truthful information with scientific support on the subject, as an ethical and professional commitment to society.

## RESUMO

**OBJETIVO:**

Analisar os riscos relacionados às vacinas e os impactos da não vacinação para a população mundial.

**MÉTODOS:**

Revisão narrativa que considerou informações contidas nas bases de dados bibliográficos NCBI-PubMed, Medline, Lilacs e *Scientific Electronic Library Online* (SciELO), no período compreendido entre novembro de 2015 e novembro de 2016. Para a análise de surtos ocasionados pela não vacinação foram considerados os trabalhos publicados entre 2010 e 2016.

**RESULTADOS:**

Foram descritos os principais componentes das vacinas oferecidas pelo sistema público de saúde brasileiro e eventos adversos associados a esses elementos. Com exceção de reações inflamatórias locais e efeitos raros como exacerbação de doenças autoimunes e alergias, não foi demonstrada relação causal entre a administração de vacinas e autismo, mal de Alzheimer ou narcolepsia. Por outro lado, a falta de informações e a divulgação de informações não científicas têm contribuído para a reemergência de doenças infecciosas em diversos países no mundo e põe em risco planos globais para a erradicação de doenças infecciosas.

**CONCLUSÕES:**

A população deve estar bem informada quanto aos benefícios da vacinação e os profissionais da saúde devem assumir o papel de divulgar informações verídicas e com respaldo científico sobre o tema, como compromisso ético e profissional junto à sociedade.

## INTRODUCTION

The first vaccine was discovered by Edward Jenner in 1796, after 20 years of studies and experiments with cowpox, giving rise to the terms vaccine and vaccination (derived from the Latin term for cow, *vacca*). In eighteenth-century England, smallpox accounted for approximately 10% of total deaths, and one-third of these deaths were among children. Classified as one of the most devastating diseases in the history of mankind, smallpox was considered eradicated by the World Health Organization (WHO) in 1980, following the implementation of a world-wide mass vaccination program^1^.

Despite the notable relevance in eradicating or controlling various infectious diseases, vaccines are often related to questioning and criticism about the adverse effects. They have also been involved in some tragic events in the pharmaceutical industry. The biggest one occurred in 1955, after failure in the manufacturing process of the inactivated poliomyelitis vaccine^2^. Other episodes were recorded involving specific components of the BCG (Bacillus Calmette-Guérin), MMR (measles, mumps, and rubella), rotavirus^3^, oral polio^4^, and cell pertussis^5^ vaccines. Because of such events, efforts have been made to ensure greater safety in the manufacture and use of vaccines and they have definitely solved problems such as those mentioned above. The inactivated formulation (known as Salk or IPV) is currently administered in children up to four months. Because they contain dead viruses, they avoid the serious adverse effects observed with the attenuated virus formulation (OPV). Another example is the pertussis vaccine (present in the DTaP vaccine – diphtheria, tetanus, and acellular pertussis), which has undergone modifications to replace the cell pertussis vaccine, related to serious adverse events in the 1970s^6^.

The creation of the National Immunization Program (PNI), in 1973, by determination of the Ministry of Health was a major step for the public health in Brazil. Currently, 19 vaccines recommended by the WHO are offered free of charge in the Brazilian Unified Health System (SUS) and they benefit all age groups, following a national vaccination schedule^7^. In order to coordinate immunization actions, the program ensured the continuity of doses (compliance with schedule) and expanded the coverage area in Brazil^8^, reaching averages above 95% of vaccine coverage for the immunization schedule of children^9^. Some important results are the elimination of poliomyelitis and the sustained transmission of measles and rubella in the country^9^.

However, despite the impact on reducing cases and deaths from vaccine-preventable diseases, anti-vaccination movements are increasingly frequent and persuasive. These movements use strategies such as distortion and dissemination of false information that, claiming to have a scientific basis, question the efficacy and safety of various vaccines^10^. Most of them relate vaccines, such as the MMR, adjuvants, and the thimerosal preservative to the occurrence of autism in children. A temporal association is sought, mainly because the disease is diagnosed after the application of most vaccines present in the immunization schedule of the child, without necessarily having a causal relationship. Another example of an association without a causal relationship recently reported in the media was the occurrence of cases of temporary paralysis following immunization with the human papillomavirus (HPV) vaccine.

Vaccines are rigorously tested and monitored by their manufacturers and the health systems of the countries where they are applied. The licensing and marketing of vaccines occur only after approval by specific regulatory agencies and careful, costly, and time-consuming clinical trials (phase I, II, and III) with accredited volunteers. Phase IV occurs only after approval of the commercialization of the product and its main objective is to detect adverse events not registered in the previous phases, the so-called Adverse Events Following Immunization (AEFI). The WHO recommended the surveillance of AEFI in 1991, and the National System for the Surveillance of Adverse Events Following Immunization (VEAPV) was structured in Brazil in 1992. In addition, the National Institute for Quality Control in Health (INCQS), directly linked to the National Health Surveillance System, ensures the quality of the immunobiologicals distributed, whose rejection rates are less than 1%^4^.

A quick search with the term “anti-vaccination” in one of the largest social networks currently used pointed to 20 pages and 17 groups related to anti-vaccination movements, with almost 15,000 followers in one of them. The same term was applied to the largest web search engine, which had more than six million results, including blogs and communities that support non-vaccination. This shows the need to clarify the population about the importance of vaccines and the danger posed by non-vaccination.

This study aimed to evaluate the possible risks associated with the vaccines in use in Brazil.

## METHODS

This is a study based on literature searches on the investigation of the risks associated with vaccination and outbreaks triggered by the practice of non-vaccination and related subjects. This is a narrative review, which considered information present in the following bibliographic databases: PubMed, Medline, Lilacs, and Scientific Electronic Library Online (SciELO). Regarding the investigation of the risks associated with vaccines and their components, the search was carried out between November 2015 and November 2016 using the descriptors: PVAE, adverse events, vaccine, vaccination, and terms related to a disease, vaccine, or specific component, such as autism, syndrome, HPV vaccine, MMR vaccine, thimerosal. The same terms, in Portuguese, were also used: “*AEFI*”, “*eventos adversos*”, “*vacina*”, “*vacinação*”, “*autismo*”, “*síndrome*”, “*vacina de HPV*”, “*vacina tríplice viral*”, “*timerosal*”. For the analysis of outbreaks caused by non-vaccination, we searched the NCBI-PubMed platform with terms related to diseases with available vaccination, for example measles and mumps, added to the terms “outbreak” and “unvaccinated”. The results were selected after reading the summary of the articles and we considered only articles that showed a clear relation between non-vaccination events and outbreaks or epidemics of the disease, published between 2010 and 2016. We carried out a survey on the subject using the terms “anti-vaccine movement”, “vaccine hesitancy”, “vaccine refusal”, and “non-vaccination” added or not to the term “outbreak”. We excluded articles, letters, abstracts, or dissertations in other languages besides Portuguese, English, or Spanish.

## RESULTS

### Risks Associated with Immunization

Most of the news linked in informal social media and some published works have presented or suggested autism or Autism Spectrum Disorder (ASD) as one of the main diseases attributed to the practice of vaccination, mainly for the measles, mumps, and rubella (MMR) vaccine. However, the Brazilian Health Surveillance Agency (ANVISA), as well as the American Food and Drug Administration (FDA) have not shown any association between vaccines and the increase of cases of autism in the population^2^ ^,^ ^11^ ^–^ ^13^.

Adjuvants, often found in vaccine formulations, may also be associated with the onset of adverse reactions and events. Among the adjuvants used in the production of vaccines, we can mention mineral salts (aluminum salts and calcium salts), microbial derivatives such as, Monophosphoryl Lipid A (MPL) and oil-in-water emulsions using squalene as main compound (AS03 and MF59)^14^. The administration of these compounds may lead to adverse reactions^8^, such as local inflammatory reactions and, much less frequently, systemic effects, such as the exacerbation of autoimmune diseases and allergies. In addition, other diseases have been investigated regarding the causal relationship with adjuvants^15^. We highlight the reactions to aluminum salts, such as allergies and ASIA (Autoimmune/Inflammatory Syndrome Induced by Adjuvants)^15^ ^,^ ^16^, macrophagic myofasciitis^15^ ^,^ ^17^, and neurological diseases, such as Alzheimer^18^ and syndromes included in the ASD^18^. However, no correlation has been reported in the scientific analyses for any of them. In addition to aluminum salts, we also highlight diseases related to squalene, present in the pandemic and seasonal influenza vaccines, such as Gulf War Syndrome^15^ ^,^ ^18^ and Narcolepsy^19^ ^,^ ^20^ (Table 1), but no causal relationship has been found.

**Table 1:** Adverse events associated with the use of vaccine adjuvants.

Adjuvant	Vaccine	Adverse events studied	References
Aluminum salts	DPT Pentavalent Hepatitis A and B Meningococcal	Macrophagic myofasciitis, Alzheimer, and ASD	17,21
	HPV	Postural Orthostatic Tachycardia Syndrome (POTS), Guillain-Barré Syndrome (GBS)	22–25
Squalene – MF59	HIV; Herpes zoster; Cytomegalovirus; Influenza	Gulf War Syndrome	8,26
Squalene – AS03	Influenza	Narcolepsy Paresthesia	8,27,28

DPT: diphtheria, tetanus, and pertussis; HPV: human papillomavirus; ASD: Autism Spectrum Disorder

In addition to the adjuvants, other vaccine components, such as stabilizers and preservatives, may be related to different adverse events (Table 1). The main examples are: albumin and gelatin (proteins used as stabilizers); antibiotics, commonly used during the early stages of vaccine preparation and often associated with allergic reactions; and formaldehyde, which in liquid form, is used in the initial stages of some vaccines as an inactivating agent for toxins or viral particles. Formaldehyde has been linked to some adverse events such as eczema and even cancer. However, studies evaluating the association of cancer with the use of formaldehyde have proved the association after exposure to large amounts or frequent exposure, that is, under conditions that do not apply to vaccines^29^ ^,^ ^30^. Egg proteins may also be present in very low amounts in some vaccines that use viruses grown in embryonated eggs, such as the influenza vaccine. These proteins may trigger an allergic response in persons intolerant to this component^31^ ^,^ ^32^.

Used as a preservative in some licensed vaccines, thimerosal is an organic compound based on mercury that has also been involved in controversial issues about vaccine safety. The association between autism, mercury, and vaccines came with the publication of a paper in 1998 by the English physician Andrew Wakefield in one of the most important scientific journals^33^. In this study, the authors pointed out symptoms, such as intestinal disorders and developmental delay in twelve children evaluated, and behavioral changes (including autism) in nine of them. In 2010, after a court decision, the article was fully removed after it was discovered the presence of false information in the study^34^, as well as payment agreements involving the researcher and attorneys in compensation cases for vaccine damages. Some studies have also shown that the dose of mercury normally ingested by an individual in the diet is much higher than the amount present in vaccines^35^ ^–^ ^38^. To date, no regulatory agency has actually proved the association between these diseases and the preservative.

In general, the occurrence of hypersensitivity reactions depends on the susceptibility, which makes the individual predisposed to its occurrence. Thus, the administration of certain vaccines is contraindicated in patients with a history of anaphylactic reaction to milk, eggs, or any other component that is present in a particular formulation. There is also evidence that some adverse events are the result of genetic factors, such as influenza vaccine-related narcolepsy (with squalene and alpha-tocopherol as adjuvant). Other adverse events are considered as idiosyncratic, that is, dependent on individual factors^8^.

Recently, prophylactic vaccines against HPV infection gained space in the media for possible involvement with the Guillain-Barré Syndrome (GBS) and Postural Orthostatic Tachycardia Syndrome (POTS). The GBS is an autoimmune disease that causes damage to the nervous system and causes tingling sensation, muscle weakness, and even paralysis (Table 2). In general, it manifests itself following vaccination with formulations containing viral vectors^4^ or, in this case, VLP (Viral-Like Particles) used in the currently marketed HPV vaccines. The POTS is also a syndrome that causes nerve damage, but it causes slightly milder symptoms such as palpitations, malaise, and dizziness (Table 2). In fact, some studies have shown a temporal association between vaccines and GBS/POTS^24^ ^,^ ^25^. However, the WHO reports that no serious adverse events have been reported even after its application to millions of people and that the occurrence of GBS in vaccinated persons has a frequency similar to cases of disease with an unknown cause^41^. The marketing of these vaccines continues to be released by ANVISA and the FDA, and any adverse effects must be reported to health agencies or to those responsible for the distribution and administration of the vaccines.

**Table 2:** Adverse events related to other vaccine components.

Component	Function in the vaccine	Vaccine	Dose limit	Adverse events studied	References
Albumin (recombinant)	Stabilizer	Varicella/MMR	50 ng/dose	Anaphylaxis	8
Antibiotics	To avoid contamination in the manufacturing process	Yellow fever, influenza, polio	NA	Allergic reactions	8
Formaldehyde/Glutaraldehyde	To inactivate toxins or viral particles	Influenza	200 ppm (residual)	Eczema, cancer	8,29
Gelatin	Stabilizer	Yellow fever	10 µL/mL	Allergy, anaphylaxis, hypersensitivity	8,31,39
Egg proteins	Virus cultivation	Yellow fever, influenza	NA	Allergy, anaphylaxis	8,32
Sorbitol	Stabilizer	Yellow fever, HPV, pneumococcal conjugate, polio	NA	Intolerance, allergy	8
Thimerosal	Preservative	DPT, MMR, Hepatitis B, influenza	0.01% or 25 µg Hg/dose	Allergy, ASD	8,35–38,40

NA: Not applicable; ASD: Autism Spectrum Disorder; HPV: human papillomavirus

Another vaccine formulation that deserves attention in relation to the possible occurrence of adverse events is the dengue vaccine, recently licensed for use in Brazil. Known as “CYD-TDV” or “Dengvaxia”, the vaccine is based on four attenuated virus strains of chimeric composition, that is, it consists of the yellow fever virus expressing envelope proteins of four DENV serotypes^42^. The formulation was submitted, in parallel, to two phase III clinical studies in Asian and Latin American countries (including Brazil), involving more than 30,000 participants aged between two and fourteen years (study “CYD14”) and nine to sixteen years (study “CYD15”) who received three doses^43^ ^,^ ^44^. The contraindications stipulated by the manufacturer are the same as those found for most attenuated vaccines and include individuals with allergies to any of the vaccine components (no adjuvants in the formulation), immunosuppressed individuals, and pregnant or lactating women. Local and systemic adverse events following immunization were similar to those reported by other live attenuated vaccines^45^.

However, the group of vaccinated individuals aged two to five years had a higher risk of hospitalization with severe forms of dengue compared to the placebo group of the same age group when infected with the wild virus in tests performed in Asia^44^. This event was observed mainly in the third year after the first dose and it was not observed in other age groups (6–8, 9–11, and 12–16 years). In addition, an overall estimate against all serotypes of the virus resulted in a final efficacy of approximately 60% but an efficacy of 33.7% for the younger group (2–5 years)^45^. Such observations suggest that vaccination may contribute with an exacerbated secondary infection in very young seronegative children – a phenomenon known as ADE (Antibody Dependent Enhancement). In this phenomenon, viral replication is increased in individuals vaccinated or infected with viral strains other than those previously exposed, from the presence of non-neutralizing antibodies. Therefore, the vaccine was only indicated for children over nine years of age.

### Risks Associated With Non-Vaccination

Among the risks related to vaccines, non-vaccination is considered the most important. The deleterious effects associated with the use of vaccines, when present and scientifically proved, occur at a very low frequency and are inexpressive when compared to risks related to non-vaccination. Strategies to stimulate the use of vaccines are traditionally adopted in public health, but they may be insufficient to ensure an increase in vaccine coverage. In this context, a clear understanding of the value of vaccines needs to be kept both in the population and among health professionals^46^. In Brazil, vaccination is mandatory and regulated by federal legislation (Decree 78,231, of August 12, 1976)^47^.

However, the non-vaccination decision is individual and influenced by factors such as public health policies, recommendation of health professionals, media, and factors intrinsic to the individual, such as knowledge and information, past experiences, perception of the importance of vaccination, and moral and religious convictions. These factors are inserted in a historical, political, and social context that should also be considered^48^. However, the decision of the individual does not only affect him or her. The decision not to vaccinate or the persuasion of persons to avoid doing so contributes to reduce population immunity (or herd immunity), which may result in localized outbreaks or pockets of infection in specific groups or populations. This type of situation has assumed worrying proportions, especially since the 1970s and 1980s, when cases of pertussis exponentially increased in developed countries, a disease that is easily controlled with adequate vaccination coverage^49^.

These outbreaks are becoming more frequent and may be related to several factors (Table 3). Most studies report individuals who individually decided not to vaccinate or who traveled or migrated from an environment with high vaccine coverage to another with low vaccine coverage, exposing unvaccinated populations to the pathogen. Because of this phenomenon, some diseases previously controlled by effective vaccination programs, such as measles, have resurfaced in populations from different parts of the world, including Brazil.

**Table 3:** Scientific articles published between 2010-2016, which correlate outbreaks of infectious diseases to unvaccinated individuals and their respective reasons/sources of outbreaks.

Disease	Country of outbreak (year)^Outbreak Reason/Source^	References
Measles	Qatar (2007)^a^; China (2010)^a^; Norway (2011)^a^; Macedonia (2010–2011)^a^; Ireland (2009–2010)^b^; Poland and Greece (2010)^b^; Serbia (2010–2011)^b^; Spain (2012)^b^; Zimbabwe (2010)^c^; USA (2013)^c^; Netherlands (2013)^c^; Spain (2010-2011)^c^; USA (2009)^d^; Norway (2011)^d^; England (2013)^d^; USA (2011)^e^; Northern Ireland (2010)^f^; Spain (2010)^f^; Israel (2012)^f^; USA (2011, 2012, 2013)^f^; England (2008–2009)^f^; USA (2008, 2011)^g^; Italy (2008, 2009, 2010)^g^; Spain (2011)^g^; China (2013)^h^; Democratic Republic of Congo (2010)^h^; Romania (2011)^h^; France (2008–2011)^h^; Brazil (2012–2013^a^, 2013–2015^h^)	54–86
Mumps	Canada (2008)^g^; USA (2011)^g^; Netherlands and Canada (2007–2009)^c^; USA (2009–2010)^c^; Bosnia Herzegovina (2010–2011)^i^; Mongolia (2011)^h^	87–92
Diphtheria	Norway (2008)^g^	93
Rubella	Romania (2011–2012)^g^; Japan (2012–2013)^g^; China (2010–2011)^a^	94,95
Pertussis	Brazil (2004, 2007)^h^; USA (2009–2010)^c^; USA (2010, 2012)^g^; Italy (2009)^h^; Papua New Guinea (2011)^j^	96–102
Yellow fever	Brazil (2008–2009^h^, 2015^a^); Angola and Democratic Republic of Congo (2015–2016)^h^	50–52,103
Poliomyelitis	Somalia (2013)^i^	104

a Migration/Immigration of unvaccinated individuals.

b Gypsy communities.

c Orthodox communities.

d Unvaccinated health professionals.

e Refugees.

f Unvaccinated travelers.

g Individuals intentionally unvaccinated.

h Vaccination failures/Accumulation of unvaccinated persons.

i Armed conflicts.

j Geographical isolation.

In relation to yellow fever, more than 100 million persons were vaccinated in West Africa in 2015. However, Angola and the Democratic Republic of Congo had an outbreak of this disease between December 2015 and January 2016. Men accounted for approximately 70% of the cases^50^ ^,^ ^51^. Studies have related this outbreak to the high population density coupled with the low vaccination coverage of men. In the same year, nine fatal cases of yellow fever were reported, five in Brazil, all in non-vaccinated persons, but in situations with vaccination recommendation (tourism or residents of rural areas)^52^. According to the Center for Emergency Operations in Public Health (COES), 371 cases and 127 deaths were recorded from 2016 to March 2017. It is speculated the outbreak relation with the low vaccination coverage (with the inclusion of non-endemic regions) and some factors such as deforestation and environmental accidents in wildlife habitats, including non-human primate hosts.

Measles has been considered as eliminated in the Americas since 2002, but it has a growing incidence in Brazil and the world, which is a reflection of voluntary non-vaccination. Between 2013 and 2015, more than 1,000 cases were reported only in the states of Pernambuco and Ceará, affecting individuals aged between 15 and 29 years (34%) and infants under one year (27.5%) from the circulation of one virus type from Europe^53^ ^,^ ^54^. According to the WHO, the estimate is that immunization has prevented more than 20 million deaths between 2000 and 2015 worldwide, making the measles vaccine one of the most effective in public health.

## FINAL CONSIDERATIONS

The risks associated with the use of available vaccines do not justify discontinuation of any available formulation in the market. On the other hand, the risk associated with “non-vaccination” causes growing concerns in several countries. Advertising campaigns, disseminated in social media or even dressed in supposedly “scientific” evidence, contribute to the resurgence of diseases once eradicated in much of the world. In Brazil, in particular, lack of information and dissemination of unfounded information contribute to the reappearance of infectious diseases, such as measles and pertussis. We also highlight the risk associated with non-acceptance of vaccines, such as those involving the prevention of HPV infection, and we can only expect its impacts on mortality if adequate conditions of vaccine administration and coverage are kept. The role of health professionals in disseminating the benefits associated with vaccination is important to ensure the health and quality of life of the population.
